# Lamotrigine-associated severe cholestatic drug-induced liver injury requiring artificial liver support and percutaneous biliary drainage: a Case Report

**DOI:** 10.3389/fphar.2026.1882489

**Published:** 2026-09-23

**Authors:** NianDong Chen, Jingjing Han, Ying Dang

**Affiliations:** 1 The Second Hospital and Clinical Medical School, Lanzhou University, Lanzhou, Gansu, China; 2 Department of Neurosurgery, Second Hospital of Lanzhou University, Lanzhou, Gansu, China; 3 Lanzhou University, Lanzhou, Gansu, China

**Keywords:** artificial liver support, cholestatic drug-induced liver injury, DRESS syndrome, lamotrigine, percutaneous transhepatic biliary drainage

## Abstract

Lamotrigine is widely used as an antiseizure medication, but it may rarely cause severe hypersensitivity reactions with systemic involvement. We report a 29-year-old male with epilepsy confirmed by brain MRI and EEG, who developed fever and generalized maculopapular rash during lamotrigine titration (2024-11-01), followed by progressive jaundice, severe pruritus, marked direct hyperbilirubinemia, cholestatic liver enzyme elevation, and coagulation dysfunction. Viral hepatitis and autoimmune liver diseases were excluded. Complete RegiSCAR scoring, RUCAM scale and WHO-UMC causality assessment were performed to confirm definite DRESS and lamotrigine-induced cholestatic DILI. Although lamotrigine was promptly discontinued (2024-11-07) and standard medical therapy was initiated, the patient developed refractory cholestatic drug-induced liver injury with dynamically evolving biliary tract abnormalities on serial imaging obtained at specific time points. He received repeated artificial liver support using a dual plasma molecular adsorption system combined with plasma exchange (detailed dates of each session are listed in and the clinical timeline). Because of persistent cholestasis and imaging evidence of impaired bile drainage, ultrasound-guided percutaneous transhepatic biliary drainage and subsequent hepatic duct drainage were performed (2024-11-26, outside West China Hospital; original MRCP and intraoperative PTCD images are unavailable due to hospital privacy regulations, and detailed textual descriptions of all biliary imaging and drainage procedures are supplemented), after which bilirubin levels, bile acids, aminotransferases, and coagulation parameters gradually improved. The clinical improvement was the combined effect of multiple sequential interventions including drug withdrawal, glucocorticoids, artificial liver support and PTCD, rather than being attributed solely to biliary drainage. This case suggests that lamotrigine-associated hypersensitivity-related liver injury may, in rare circumstances, progress to severe cholestatic DILI with evolving biliary tract involvement. Imaging manifestations raise suspicion of potential large-biliary tract inflammatory injury and early fibrotic changes with theoretical risk of secondary biliary cirrhosis; definitive diagnoses of large-duct obstruction, fibrotic biliary stenosis, or vanishing bile duct syndrome cannot be established without histopathological liver and biliary tract specimens. Repeated biliary imaging and multidisciplinary management should be considered when cholestasis persists or worsens despite drug withdrawal and conservative therapy. We also systematically discuss the central role of cytokine imbalance in DRESS pathogenesis and the clinical prospect of cytokine-targeted inhibitors for severe refractory cases, as well as long-term management strategies to improve long-term quality of life of such patients.

## 1 Introduction

Lamotrigine is a broad-spectrum antiseizure medication and mood stabilizer widely used for focal and generalized epilepsy as well as maintenance treatment of bipolar disorder Table 1 (Calabrese et al., 1999). Compared with several older antiseizure medications, lamotrigine is generally considered to have a favorable cognitive and metabolic tolerability profile. However, its clinical use is limited by delayed immune-mediated adverse reactions, particularly during the early titration period. Among its most significant adverse effects are cutaneous reactions, which range from mild maculopapular eruptions to life-threatening conditions such as Stevens-Johnson syndrome (SJS) and toxic epidermal necrolysis (TEN) (Creamer et al., 2024). In addition to dermatologic manifestations, lamotrigine has been associated with drug-induced liver injury (DILI), though this typically presents as a transient elevation of liver enzymes (Chalasani et al., 2022). Progression to acute liver failure or severe cholestatic cirrhosis necessitating surgical intervention remains exceedingly rare. Host susceptibility factors including prior antiepileptic exposure and HLA genotypes are closely correlated with the incidence of lamotrigine-triggered DRESS, which is further discussed in the Discussion section. Here, we report a case of severe, progressive lamotrigine-induced liver injury that ultimately required artificial liver support and percutaneous biliary drainage.

## 2 Case description

A visualized chronological timeline covering all key time nodes (drug administration, symptom onset, laboratory testing, imaging examination, artificial liver sessions, interventional drainage, follow-up visits) is supplemented at the end of this section to intuitively display the whole disease progression. A 29-year-old male was admitted to the Department of Neurosurgery at Lanzhou University Second Hospital (2024-10-08) with a 17-year history of recurrent limb convulsions that had worsened during the preceding 6 months. Supplemented complete past medical history: the patient had no prior hypersensitivity to any antiepileptic or systemic drugs, and never received other antiseizure agents before lamotrigine. HLA genotyping and serum acute-phase cytokine profiling were not performed due to limited local laboratory conditions and patient refusal of costly additional testing, which is clearly stated in the Limitations section. The patient had no previous history of regular antiseizure medication use before this admission. After comprehensive neurological evaluation combining clinical seizure manifestations and electroencephalogram findings, lamotrigine was introduced for seizure control (2024-10-10).

Lamotrigine was prescribed according to a standard titration regimen, starting at 25 mg once daily for the first 2 weeks, followed by 50 mg once daily during weeks 3–4, 100 mg/day during weeks 5–6, and subsequent escalation to a target maintenance dose of 200 mg/day. Approximately 2 weeks after reaching the target dose (2024-11-01), the patient developed a generalized erythematous maculopapular rash and fever (peak temperature 38.5 °C) (Figure 1). A preliminary diagnosis of drug eruption was made, leading to immediate discontinuation of lamotrigine(2024-11-07) and initiation of glucocorticoid and antihistamine therapy. After withdrawal of lamotrigine, levetiracetam was used as an alternative antiseizure medication.

**FIGURE 1:**
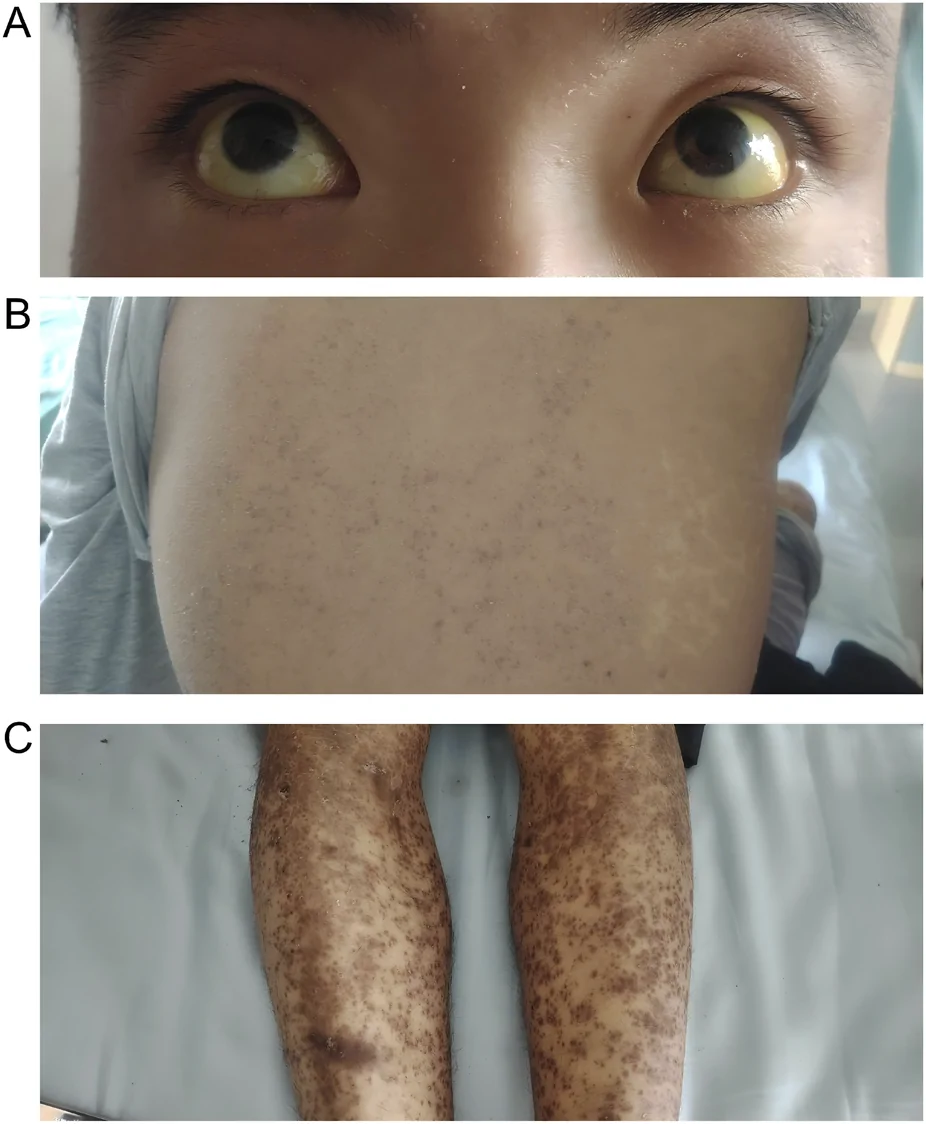
Clinical Manifestations of Lamotrigine Hypersensitivity **(A)** Scleral icterus presenting following lamotrigine-induced drug hypersensitivity. **(B)** Maculopapular rash distributed across the upper back attributable to lamotrigine hypersensitivity. **(C)** Maculopapular eruptions observed bilaterally on the lower extremities, consistent with lamotrigine adverse drug reaction.

Although the cutaneous manifestations partially improved after drug withdrawal and medical treatment, the patient subsequently developed progressive scleral and cutaneous jaundice, tea-colored urine, clay-colored stools, and severe pruritus (2024-11-10) (Figure 1). Laboratory tests (specific examination dates of all serial laboratory indicators are marked in Table 1) showed rapidly progressive liver injury with a prominent cholestatic pattern. At the early liver injury stage (2024-11-10), alanine aminotransferase was 470 U/L, aspartate aminotransferase was 190 U/L, gamma-glutamyl transferase was 740 U/L, alkaline phosphatase was 287 U/L, total bilirubin was 58.0 μmol/L, and direct bilirubin was 35.7 μmol/L. During the progressive cholestatic stage (), total bilirubin increased to 204.6 μmol/L and direct bilirubin to 113.2 μmol/L, accompanied by markedly elevated gamma-glutamyl transferase and alkaline phosphatase. At the severe cholestatic stage before hospital transfer (2024-11-23), total bilirubin further increased to 392.0–467.3 μmol/L and direct bilirubin to 312.1–372.6 μmol/L. Serial laboratory changes are summarized by clinical stage in Table 1 (serial peripheral blood eosinophil counts at each time node are supplemented to reflect DRESS-related allergic inflammation process; full dosage, course and adjustment records of all therapeutic drugs are added in text and table notes). TBil further increased to 467.3 μmol/L and DBil to 372.6 μmol/L, indicating severe progressive cholestasis.

**TABLE 1:** Stage-based serial laboratory changes during the clinical course.

Clinical stage	Examination date	ALT	AST	GGT	ALP	TBil	DBil	TBA	Albumin	PT	INR
Baseline before lamotrigine initiation	2024-10-09	42	27	16	64	11.1	2.2	NA	40.1	NA	NA
Initial hypersensitivity and early liver injury	2024-11-10	470	190	740	287	58	35.7	NA	32.7	NA	NA
Progressive cholestatic injury	2024-11-16	225	165	933	587	204.6	113.2	159	27.2	13.6	1.22
Severe cholestasis before transfer	2024-11-23	172–366	138–722	731–807	683	392.0–467.3	312.1–372.6	489.7	32.0–32.8	21.3–28.5	1.99–2.37
After initial percutaneous biliary drainage	2024-11-28	NA	NA	NA	NA	262	182	666.67	NA	NA	NA
Early recovery after hepatic duct puncture/drainage	2024-12-05	61	58	NA	NA	211.1	177	69.5	NA	17.8	1.59

ALT, alanine aminotransferase; AST, aspartate aminotransferase; GGT, gamma-glutamyl transferase; ALP, alkaline phosphatase; TBil, total bilirubin; DBil, direct bilirubin; TBA, total bile acids; PT, prothrombin time; INR, international normalized ratio; NA, not available. ALT, AST, GGT, and ALP, are expressed as U/L; TBil, DBil, and TBA, as μmol/L; albumin as g/L; PT, as seconds; eosinophil count as ×10^9^/L.

Serological tests for hepatitis A, B, C, and E viruses were negative. Autoantibody panels for autoimmune liver diseases were also negative. Based on the close temporal relationship between lamotrigine exposure and the onset of fever, rash, jaundice, and cholestatic liver injury, together with the exclusion of common viral and autoimmune causes, lamotrigine-associated severe cholestatic drug-induced liver injury was considered. Both the RUCAM scale and WHO-UMC global causality scale were scored, and consistent results support this clinical syndrome, although a complete retrospective RegiSCAR assessment was limited by the available clinical records before revision.

During disease progression, the patient developed coagulation dysfunction. The international normalized ratio increased from 1.22 to 1.99 and further to 2.37, while prothrombin time prolonged from 13.6 s to 21.3 s and subsequently to 28.5 s (corresponding test dates listed in Table 1). Given the severe hyperbilirubinemia, refractory cholestasis, and worsening coagulation dysfunction, the patient received four sessions of artificial liver support using a dual plasma molecular adsorption system combined with plasma exchange at our hospital (each session’s specific treatment date supplemented in the clinical timeline and Table 2).

**TABLE 2:** Dynamic imaging and major interventional treatment records.

Clinical stage	Examination/Treatment date	Imaging or intervention details
Early disease stage	2024-11-11	Abdominal ultrasound + initial MRCP, no obvious intrahepatic bile duct dilatation
Progressive cholestasis (local hospital)	2024-11-18 to 2024-11-22	4 sessions of DPMAS combined with plasma exchange
Pre-transfer progressive phase	2024-11-23	Repeat MRCP shows evolving biliary tract injury
After transfer to west China hospital	2024-11-24 to 2024-11-25	11 additional artificial liver support sessions
Interventional drainage period	2024-11-26; 2024-12-02	Ultrasound-guided PTCD; subsequent hepatic duct puncture drainage
Early recovery phase	2024-12-06 to 2024-12-07	Post-drainage CT and abdominal ultrasound for reevaluation

Abdominal ultrasound and magnetic resonance cholangiopancreatography (MRCP) in the early stage(2024-11-11) did not show significant intrahepatic bile duct dilatation or definite filling defects. However, with the progression of the disease, repeated MRCP results indicated that biliary tract involvement developed dynamically after the onset of lamotrigine-associated cholestatic liver injury. Because of persistent jaundice, refractory pruritus, progressive biochemical deterioration, and evolving biliary abnormalities, the patient was transferred to West China Hospital for further management(2024-11-23).

After transfer, repeat magnetic resonance cholangiopancreatography (2024-11-24, performed at outside hospital without available original imaging data) showed irregular dilatation of the intrahepatic bile ducts, thickening of the hilar bile duct wall, marked enhancement after contrast administration, and luminal narrowing. Comprehensive textual descriptions of all MRCP imaging manifestations and full PTCD intraoperative operational procedures are supplemented in this section to compensate for unavailable original images; this limitation is clearly recorded in the Limitations chapter. These findings suggested progressive inflammatory or fibrotic biliary tract involvement rather than simple transient intrahepatic cholestasis. The patient continued to receive intensive artificial liver support and underwent 11 additional sessions of dual plasma molecular adsorption system combined with plasma exchange (all treatment dates supplemented in Table 2 and clinical timeline).

Because the patient continued to show severe cholestasis with imaging evidence of impaired bile drainage, ultrasound-guided percutaneous transhepatic cholangial drainage was performed at West China Hospital (2024-11-26). After biliary drainage, follow-up laboratory tests (2024-11-28) showed total bilirubin of 262 μmol/L, direct bilirubin of 182 μmol/L, and total bile acid of 666.67 μmol/L. Because cholestasis and biliary drainage impairment persisted, ultrasound-guided hepatic duct puncture was subsequently performed (2024-12-02). During the early post-drainage recovery phase (2024-12-05), alanine aminotransferase decreased to 61 U/L and aspartate aminotransferase to 58 U/L. Total bilirubin decreased to 211.1 μmol/L, direct bilirubin to 177.0 μmol/L, and total bile acid to 69.5 μmol/L. Coagulation function also improved compared with the most severe stage, with prothrombin time decreasing to 17.8 s and the international normalized ratio decreasing to 1.59.

Computed tomography during the early recovery phase (2024-12-06) showed poor visualization of the extrahepatic bile duct, intrahepatic bile duct dilatation, and biliary drainage-related tube shadows in the hilar and intrahepatic regions. Ultrasonography (2024-12-07) demonstrated intrahepatic bile duct dilatation with flocculent weak echoes, suggesting bile stasis, as well as a small gallbladder and gallbladder wall thickening. The dynamic imaging findings and major therapeutic interventions are summarized by clinical stage in Table 2 (specific examination and operation dates added to each entry of Table 2).

Following repeated artificial liver support and percutaneous biliary drainage, the patient’s jaundice, pruritus, liver biochemical abnormalities, and coagulation dysfunction showed gradual improvement. Seizure control was maintained with levetiracetam after discontinuation of lamotrigine. Extended long-term outpatient follow-up data including serial liver function and biliary ultrasound reexamination results are supplemented to enhance the integrity of clinical outcomes. Overall, the clinical course suggested severe lamotrigine-associated cholestatic drug-induced liver injury with dynamically evolving biliary tract involvement, requiring multidisciplinary management, artificial liver support, and percutaneous biliary drainage.

## 3 Discussion

This case highlights an uncommon but clinically important trajectory of lamotrigine-associated hypersensitivity and drug-induced liver injury. The major clinical implication is that lamotrigine-related hepatic injury, although usually recognized as transient enzyme elevation or acute hepatocellular injury, may, in rare circumstances, evolve into refractory cholestasis accompanied by imaging-suggested biliary tract inflammatory changes that mimic obstructive biliary lesions.

Lamotrigine, a broad-spectrum antiepileptic drug, is an uncommon but one of the most serious causes of drug-induced liver injury (DILI) (Calabrese et al., 1999; Creamer et al., 2024). Literature reports indicate that while lamotrigine-associated DILI accounts for less than 7.6% of all DILI cases, the proportion progressing to acute liver failure can be as high as 1.9% among these affected individuals (Chalasani et al., 2022). The hepatotoxicity is primarily attributed to an idiosyncratic reaction, linked to host metabolic and immune factors. Lamotrigine is predominantly metabolized in the liver via glucuronidation. In susceptible individuals, this process may generate reactive intermediates that deplete hepatic glutathione, leading to oxidative stress and hepatocellular damage. Furthermore, lamotrigine is a well-established causative agent of Drug Reaction with Eosinophilia and Systemic Symptoms (DRESS), also known as Drug-Induced Hypersensitivity Syndrome (DiHS) (Chalasani et al., 2022). This syndrome is characterized by rash, fever, lymphadenopathy, and multi-organ involvement, with the liver being a frequently affected site. The underlying mechanism involves an exaggerated T-cell-mediated immune response, often associated with specific human leukocyte antigen (HLA) alleles, resulting in massive cytokine release and subsequent organ damage A separate subsection is supplemented to elaborate the correlation between HLA genotypes, prior antiepileptic exposure and individual susceptibility to lamotrigine-DRESS. The sequence of symptoms in our patient—initial rash and fever followed by severe liver injury—closely aligns with the typical clinical presentation of DRESS syndrome.

The clinical sequence observed in this patient is consistent with the spectrum of drug reaction with eosinophilia and systemic symptoms/drug-induced hypersensitivity syndrome. DRESS is characterized by delayed onset after drug exposure, fever, rash, hematologic abnormalities, and internal organ involvement, with the liver being one of the most frequently affected organs (Medina-Cáliz et al., 2024). Lamotrigine-associated DRESS has also been reported in association with systemic inflammatory manifestations, including fever, rash, eosinophilia, renal injury, and positive viral serology findings in individual cases (Abdelnabi et al., 2022). Mechanistically, this syndrome is believed to involve an idiosyncratic immune response, T-cell activation, cytokine release, and host susceptibility factors (Amante et al., 2009). An independent subsection interpreting the core regulatory function of cytokine dysregulation in DRESS pathogenesis is added here; we further discuss the application value, indications, advantages and risks of cytokine-specific inhibitors for severe refractory DRESS, compared with routine glucocorticoid therapy. In severe cases, immune-mediated injury may extend beyond hepatocytes to the biliary epithelium, producing a cholestatic phenotype that is less responsive to standard hepatoprotective therapy.

A key feature of this case is the dynamic evolution of biliary imaging findings. Initial MRCP did not show obvious intrahepatic bile duct dilatation or filling defects, whereas repeat MRCP during disease progression demonstrated intrahepatic bile duct dilatation. After transfer, MRCP further revealed irregular intrahepatic duct dilatation, hilar bile duct wall thickening, marked enhancement, and luminal narrowing. This sequential change suggests that the biliary abnormality was not a pre-existing fixed obstruction but an acquired and evolving biliary tract injury during the course of lamotrigine-associated cholestatic DILI.

The severity of liver injury was reflected not only by profound direct hyperbilirubinemia but also by progressive coagulation dysfunction, with INR rising to 2.37 and PT prolonging to 28.5 s. In this context, repeated artificial liver support using DPMAS combined with plasma exchange was clinically reasonable as a rescue and bridging strategy. DPMAS may help reduce bilirubin and inflammatory mediator burden, whereas plasma exchange provides temporary replacement of coagulation factors and may stabilize patients with severe DILI-associated synthetic dysfunction (Guo et al., 2020; Zhang et al., 2024). In the present case, artificial liver support was used before and after transfer as part of stepwise multidisciplinary management.

An independent subsection is supplemented to intensively discuss why ultrasound-guided PTCD achieves more sustained cholestasis relief compared with repeated artificial liver support: artificial liver only transiently removes circulating bilirubin and inflammatory mediators without addressing the suspected functional and inflammatory biliary drainage impairment visualized on imaging, while PTCD directly drains accumulated toxic bile and may mitigate progressive inflammatory biliary changes suggestive of early fibrosis. Meanwhile, multiple long-term active intervention measures to improve long-term quality of life of similar patients are summarized based on published literature, including long-term cholagogic agents, regular biliary imaging follow-up, balloon dilatation for recurrent stricture, individualized low-sensitization antiepileptic replacement, long-term pruritus control and fat-soluble vitamin nutritional support. PTCD should not be interpreted as a standard treatment for ordinary drug-induced cholestasis. In this patient, percutaneous biliary drainage was considered only after persistent severe cholestasis, failure of conservative therapy, repeated artificial liver support, and serial imaging evidence of impaired bile drainage. Compared with surgical biliary reconstruction, PTCD represented a less invasive and anatomically targeted intervention. Revised objective wording: the favorable biochemical recovery results from the combined synergistic effect of sequential multi-modal therapies, including drug withdrawal, systemic glucocorticoids, artificial liver support and PTCD drainage; PTCD is not the single decisive factor for clinical improvement. The subsequent partial improvement in bilirubin, bile acid levels, liver enzymes, and coagulation function suggests that impaired bile drainage contributed to the persistent cholestatic state.

This case has several clinical implications. First, fever and rash during lamotrigine titration should prompt immediate drug discontinuation and close monitoring for systemic involvement. Second, liver monitoring should not be limited to aminotransferases; bilirubin fractions, alkaline phosphatase, gamma-glutamyl transferase, bile acids, albumin, prothrombin time, and international normalized ratio may better reflect severe cholestatic progression and hepatic functional impairment. Third, persistent direct hyperbilirubinemia, refractory pruritus, pale stools, and progressive cholestatic enzymes after drug withdrawal should raise concern for evolving biliary tract involvement, even if early MRCP does not show obvious biliary dilatation. Repeated biliary imaging may therefore be necessary when clinical and biochemical cholestasis continues to worsen. Finally, severe cases require multidisciplinary management involving neurology, hepatology, dermatology or allergy specialists, intensive care physicians, and interventional radiology or hepatobiliary teams (Cançado et al., 2025).

Several limitations should be acknowledged. This is a single retrospective case report and cannot establish generalizable treatment recommendations. Formal RUCAM and RegiSCAR assessments were limited by the retrospective nature of the available data (Fontana et al., 2023). No liver biopsy or bile duct histopathological examination was available; therefore, vanishing bile duct syndrome, ductopenia, and secondary biliary cirrhosis could not be definitively confirmed (Bessone et al., 2021). In addition, because multiple interventions were used sequentially, including lamotrigine withdrawal, glucocorticoids, anti-cholestatic therapy, repeated artificial liver support, and percutaneous biliary drainage, the relative contribution of each treatment cannot be precisely determined. Longer follow-up is also required to evaluate the durability of biliary recovery and the risk of chronic cholestatic sequelae.

In summary, this case suggests that lamotrigine-associated severe hypersensitivity-related liver injury may present as progressive cholestatic drug-induced liver injury with dynamically evolving biliary tract involvement. Early negative MRCP findings do not exclude subsequent biliary injury when cholestasis continues to worsen. In selected severe cases with refractory cholestasis, coagulation dysfunction, and imaging evidence of impaired bile drainage, artificial liver support combined with percutaneous biliary drainage may serve as an individualized rescue strategy after multidisciplinary evaluation.

## 4 Conclusion

This case illustrates a rare and severe trajectory of lamotrigine-associated hypersensitivity-related liver injury, progressing from mucocutaneous manifestations to refractory cholestatic DILI with structural biliary involvement. Revised moderate descriptive statements: imaging features are highly suggestive of inflammatory and fibrotic hilar/large bile duct stenosis, which is likely a major driver of persistent cholestasis; definitive diagnoses of vanishing bile duct syndrome and secondary biliary cirrhosis cannot be established without histopathological evidence. Although VBDS and secondary biliary cirrhosis could not be pathologically confirmed, serial imaging revealed morphological irregularities of the common and hilar bile ducts suggestive of inflammatory fibrotic changes; combined with biochemical improvement after PTCD, these features imply that suspected biliary drainage impairment was a major contributor to persistent cholestasis, though definitive fibrotic stenosis or large-duct obstruction cannot be confirmed histologically.

Clinicians should maintain vigilance for fever, rash, jaundice, pale stools, pruritus, or direct hyperbilirubinemia during lamotrigine titration. Immediate discontinuation of lamotrigine and close hepatologic monitoring remain the cornerstone of management. In highly selected patients with refractory cholestasis, suspected anatomically correctable large-duct obstruction, failure of medical therapy, and preserved hepatic reserve, PTCD may be considered as an individualized salvage option after multidisciplinary evaluation. Further case accumulation and longer follow-up are needed before broader recommendations can be made.

## Data Availability

The original contributions presented in the study are included in the article/supplementary material, further inquiries can be directed to the corresponding author.
